# Deciphering the pharmacological mechanisms of Weiweisu decoction in chronic atrophic gastritis: Insights from network pharmacology, molecular dynamics, and in vivo validation

**DOI:** 10.1097/MD.0000000000045348

**Published:** 2025-11-07

**Authors:** Meng Chen, Jie Liu, Shenglan Wu, Xiaoying Zhu, Yubo Jin, Yan Dang, Jialin Shen, Hua Chen, Jialin Xu, Caiqun Bie

**Affiliations:** a Guangzhou University of Chinese Medicine, Guangzhou, China; b Shenzhen Clinical College of Integrated Chinese and Western Medicine, Guangzhou University of Chinese Medicine, Shenzhen, Guangdong, China; c Shaanxi University of Chinese Medicine, Xianyang, China; d Guangzhou Medical University, Guangzhou, China.

**Keywords:** chronic atrophic gastritis, ferroptosis, in vivo validation, network pharmacology, Weiweisu decoction

## Abstract

**Background:**

This study aims to identify the phytochemical components of Weiweisu (WWS) decoction, evaluate its therapeutic potential for chronic atrophic gastritis (CAG), and elucidate the underlying mechanisms with a focus on antiferroptotic effects.

**Methods:**

Liquid chromatography–mass spectrometry was used to characterize the phytochemical constituents of WWS. Network pharmacology was applied to predict potential therapeutic targets and signaling pathways. Core compounds and targets were verified through molecular docking and molecular dynamics simulations. Subsequently, a CAG rat model was established. Enzyme-linked immunosorbent assay was used to measure levels of prostaglandins (pepsinogen I/II) and tumor necrosis factor-α in gastric tissue, while Fe^2+^, glutathione, and malondialdehyde concentrations were assessed. Gene and protein expression levels of key targets were analyzed using quantitative real-time polymerase chain reaction and western blotting.

**Results:**

A total of 1017 chemical constituents were identified via liquid chromatography–mass spectrometry. Network pharmacology revealed 20 ferroptosis-related genes associated with WWS and CAG, primarily involving cancer-related signaling pathways. In vivo experiments showed that WWS ameliorated gastric ulcers and mucosal atrophy, downregulated pepsinogen I/II and tumor necrosis factor-α levels, reduced Fe^2+^ and malondialdehyde concentrations, and increased glutathione levels. WWS treatment also elevated HSP90AA1, MAPK, and mTOR expression while inhibiting GPX4 expression. Molecular docking indicated strong binding affinities between key active compounds and core targets. Molecular dynamics simulations confirmed the stability of these interactions.

**Conclusion:**

In summary, WWS decoction can treat CAG through antiferroptosis, and its main mechanism may be related to the regulation of tumor signaling pathways.

## 1. Introduction

Chronic atrophic gastritis (CAG) is closely associated with gastric cancer (GC) and is a common gastroenterological condition. Its annual global incidence is 10.9%, and this rate continues to rise each year.^[1]^ The clinical manifestations of CAG usually include upper abdominal distension, belching, loss of appetite, acid reflux, hiccups and other digestive symptoms. Since the symptoms of patients with CAG are not specific, they are often ignored, and the risk of precancerous lesions is extremely high. At present, the principles of Western medicine treatment for CAG usually include eradication of *Helicobacter pylori* (HP) bacteria, protection of the gastric mucosa from damage and restoration of gastrointestinal function, but these treatments are often unsatisfactory. Traditional Chinese medicine (TCM) offers a complementary approach, with demonstrated benefits in slowing the related disease.^[2]^

In recent years, in-depth studies of ferroptosis have been conducted. We found that the pathogenic mechanism of CAG is associated with ferroptosis, which is explored further in this study. Ferroptosis is distinct from other known patterns of cell death, such as apoptosis and necroptosis. Ferroptosis is characterized by iron ion accumulation and iron-dependent lipid peroxidation. Moreover, the accumulation of lipid hydroperoxides plays a key role in the execution of ferroptosis.^[3]^ In addition, iron ions that accumulate during ferroptosis can produce many reactive oxygen species, which promote the accumulation of lipid hydroperoxides.^[4]^ Therefore, elucidating the molecular mechanism and regulatory network of ferroptosis can aid in the exploration of disease pathogenesis and the development of related treatments. Several relevant studies have shown that ferroptosis plays an important role in the occurrence and development of a variety of diseases, such as neurological diseases, gastroenterology-related diseases, and some kidney diseases.^[5–7]^ Moreover, ferroptosis is involved in the pathogenesis of some malignant tumors.^[8,9]^ Ferroptosis may also be involved in CAG, and inducing ferroptosis in GC cells may become a potential strategy for the treatment of CAG.

Weiweisu (WWS) decoction is a TCM composed of multiple components, making the study of its pharmacological targets and mechanisms more complex. This formulation is derived from the classic prescription Liujunzi decoction described in “Medical Biography.” Its primary function is to invigorate qi and strengthen the spleen, and it is primarily used to address spleen and stomach qi deficiency. Relevant studies have shown that Liujunzi decoction has yielded positive results in the treatment of CAG.^[10,11]^ However, a review of the literature reveals that most studies rely heavily on TCM theories and often lack modern pharmacological research.

This study intends to achieve the following goals: detect the main components in WWS; use network pharmacology to explore the ferroptosis targets and potential mechanisms of action related to WWS treatment of CAG; preliminary verification through molecular docking and molecular dynamics simulation; and construct a CAG rat model for relevant experiments to verify the potential mechanism of WWS treatment of CAG.

## 2. Materials and methods

All the databases and software versions mentioned in this study are shown in Table S1, Supplemental Digital Content, https://links.lww.com/MD/Q448.

### 2.1. Liquid chromatography-mass spectrometry/MS

#### 2.1.1. Sample preparation

WWS decoction consists of *Codonopsis pilosula* (Franch.) Nannf.of *Campanulaceae, Atractylodes macrocephala* Koidz.of *Compositae, Poria cocos* (Schw.) Wolf of *Rubiaceae, Scleromitrion diffusum* (Willd.) R.J.Wang of *Rubiaceae, Scutellaria barbata* D.Don of *Lamiaceae, Curcuma longa* L. of *Zingiberaceae, Anemarrhena asphodeloides, Pinellia ternate* (Thunb.) Makino of *Zingiberaceae, Aralia nudicaulis* L. of *Araliaceae*, and *Dioscorea oppositifolia* L. of *Dioscoreaceae* in the ratio of 1:1:1:1:1:1:1:3:3:3 and was purchased from Shenzhen Hospital of Integrated Traditional Chinese and Western Medicine of Guangzhou University of Chinese Medicine. The medicinal materials were soaked in distilled water (1:13, w/v) for 30 minutes, decocted at 100°C, extracted twice, mixed, and concentrated to obtain a Chinese herbal decoction with a crude drug content of 2.5 g/mL.

#### 2.1.2. LC-MS/MS conditions

Qualitative identification was performed using a Vanquish UHPLC system (Thermofisher, consisting of a binary pump, vacuum degasser, autosampler, and column oven) and a *Q* Exactive plus mass spectrometer (Thermofisher). The chromatographic column was ACQUITY UPLC HSS T3 (100 × 2.1 mm, 1.7 μm, waters), with a flow rate of 0.4 mL/min, a column temperature of 45℃, and an injection volume of 1 μL. The mobile phase conditions were *A*: water containing 0.1% formic acid, *B*: methanol containing 0.1% formic acid, with a gradient of *A*/*B* 95/5 to 0/100, with gradient elution within 14 minutes and 0/100 maintained for 14 to 18 minutes. Full scan and multiple reaction monitoring were performed on the samples in positive and negative ion modes. The scanning mode was DDA mode, and 10 MS/MS scans were performed after 1 full scan. The collision energy was NEC 15, 30 to fragment the ions. Nitrogen (99.999%) was used as collision-induced dissociation gas. The full scan range was 133.4 to 2000 m/z; the electrospray ionization source temperature was maintained at 320°C. The pressures of ion source gases 1 and 2 were 32 and 28 psi.

### 2.2. Network pharmacology

#### 2.2.1. Identification of the active ingredients of Weiweisu decoction

The active ingredient information of the WWS decoction was obtained from the Traditional Chinese Medicine Systems Pharmacology Database and Analysis Platform (TCMSP), the TCM Integrated Database and the HERB database (the detailed compound information is provided in Table S2, Supplemental Digital Content, https://links.lww.com/MD/Q448). The active ingredients of the WWS decoction were searched with keywords such as *C pilosula* (Franch.) Nannf. (*Campanulaceae*), *A macrocephala* Koidz. (*Asteraceae*), *P cocos* (Schw.) Wolf, *S diffusum* (Willd.) R.J.Wang (*Rubiaceae*), *S barbata* D.Don (*Lamiaceae*), *C longa L. (Zingiberaceae*), *A asphodeloides, P ternate* (Thunb.) Makino, *A nudicaulis* L. (*Araliaceae*) and *D oppositifolia* L. (*Dioscoreaceae*; the above plant name has been checked with http://mpns.kew.org,2024/11/19). The active ingredients were initially screened, the bioavailability was set at ≥30%, the drug-likeness was set at ≥0.18, and the corresponding results were obtained. After screening, the protein target information of the compounds was normalized via the UniProt database.^[12,13]^

#### 2.2.2. Screening of CAG and FRGs targets

Using “chronic atrophic gastritis” as the keyword, potential targets for the treatment of CAG in the GeneCards database and Online Mendelian Inheritance in Man database were mined.^[14]^ Using “ferroptosis” as the keyword, we mined ferroptosis-related targets in the GeneCards database and the NCBI database.

#### 2.2.3. Construction of the “herbs-active ingredients-common targets” network

The herbs, their active ingredients, and the disease-herb common targets were added to a network file and imported into Cytoscape 3.9.0 software to construct a visual “herbs-active ingredients-common targets” network. The CytoHubba plugin in Cytoscape software was used to calculate 4 topology parameters of the nodes in the network: maximum neighborhood component, density of maximum neighborhood component, maximal clique centrality, and degree. The higher the value for each parameter is, the greater the significance of the node in the network. Only the top 5 target nodes whose 4 parameters were all higher than the corresponding medians and average values were retained, and these nodes represented the key active ingredients that played a major role in the process of treatment.

#### 2.2.4. Construction of the protein–protein interaction (PPI) network

The STRING website was used to construct a protein–protein interaction (PPI) network and analyze the common targets. The analysis results were imported into Cytoscape 3.9.1 software to construct a visual network map. The Network Analyzer function of Cytoscape software was used to analyze the network. The denser the connection is, the greater the effect.^[15]^

#### 2.2.5. GO and KEGG enrichment analyses

The common targets were imported into Metascape and DAVID, the species was set to human, and *P* < .01 was used as the screening criterion. We analyzed gene ontology (GO) terms that were grouped into 3 categories, namely, biological process, cellular component, and molecular function. The same approach was used to identify enriched Kyoto encyclopedia of genes and genomes (KEGG) signaling pathways, and the microbial information online platform was used to construct a map.^[16]^

#### 2.2.6. Molecular docking analysis

The main active ingredients of the WWS decoction, lignoceric acid, formononetin, baicalein, kaempferol, and 7-methoxy-2-methyl isoflavone, which had the highest degree values in the aforementioned PPI network, were molecularly docked with the core targets epidermal growth factor receptor (EGFR), HSP90AA1, mammalian target of rapamycin (mTOR), androgen receptor (AR), and mitogen-activated protein kinase (MAPK)14. The macromolecular structures of the core targets were obtained from the RCSB Protein Data Bank; the 3D structures of the small-molecule compounds were obtained from the TCMSP database. The target proteins were dehydrated and immobilized using the PyMol tool and then imported into AutoDock Tools. Ligands and proteins were inputted into AutoDock Tools, and the target proteins were hydrated, modified with amino acids, and charge optimized. Finally, molecular docking was performed, and the results were visualized via PyMol software.

#### 2.2.7. Molecular Dynamics Simulation

The YASARA software was used to perform a 100 nanosecond molecular dynamics simulation of the optimal docking complex. This simulation is intended to further verify the binding stability between the ligand and the protein and ensure the reliability of the docking results. The simulation results help evaluate the binding affinity and stability of the complex over time, thereby gaining a deeper understanding of the interactions and mechanisms involved.

### 2.3. Animal experiments

#### 2.3.1. Animals

A total of 45 SPF Wistar male rats weighing 150 to 200 g were purchased from Guangdong Weilitong Lihua Laboratory Animal Technology. This study was approved by the Laboratory Animal Ethics Committee of the Hong Kong University of Science and Technology Medical Center, Peking University, Shenzhen (2024-210).

#### 2.3.2. Drugs

The WWS decoction consisted of *C pilosula* (Franch.) Nannf. (*Campanulaceae*), *A macrocephala* Koidz. (*Asteraceae*), *P cocos* (Schw.) Wolf, *S diffusum* (Willd.) R.J.Wang (*Rubiaceae*), *S barbata* D.Don (*Lamiaceae*), *C longa* L. (*Zingiberaceae*), *A asphodeloides, P ternate* (Thunb.)Makino, *A nudicaulis* L. (*Araliaceae*) and *D oppositifolia* L. (*Dioscoreaceae*), exist at 1:1:1:1:1:1:1:3:3:3,aggregating nearly 172 components and was purchased from Shenzhen Hospital of Integrated Traditional Chinese and Western Medicine of Guangzhou University of Chinese Medicine. The medicinal materials were soaked in distilled water (1:13, w/v) for 30 minutes, then decocted in water at 100°C, extracted twice, mixed, concentrated to obtain a Chinese medicine decoction with a crude drug content of 2.5 g/mL, and stored in 50 mL centrifuge tubes at 4°C. Folic acid tablets were purchased from Shenzhen Hospital of Integrated Traditional Chinese and Western Medicine.

#### 2.3.3. CAG model establishment, grouping and drug intervention

The rats were artificially randomly divided into 6 normal groups and 35 modeling groups via the random number table method. The modeling group of rats was generated via a 4-factor combination method that took carcinogen induction (*N*-methyl-*N*′-nitro-*N*-nitrosoguanidine, Shanghai Jizhi Biochemical Technology Co., Ltd., Shanghai,China, batch number M466338AF), gastric acid inhibitors, anhydrous ethanol (batch number 100092683), etc, into account. *N*-methyl-*N*′-nitro-*N*-nitrosoguanidine (150 μg/mL) was added to the drinking water (the drinking bottle was wrapped in aluminum foil to prevent light penetration). Additionally, a 0.05% ranitidine containing feed was administered, and different models of hunger and satiety disorders, such as a hunger and satiety disorder model of 2 days of feeding and 1 day of fasting, for a total of 16 weeks, were established. After the models were established, 5 model group rats were randomly selected to evaluate the morphology of the gastric mucosal tissue, and the model group rats were randomly split into the model group, folic acid group, the high-dose Weiweishu decoction group, the medium-dose Weiweishu decoction group, and the low-dose Weiweishu decoction group, with 6 rats in each group. With respect to the dose conversion method between humans and animals, rats were given 1/6 of the doses recommended for humans. The folic acid group was given a 2.4 mg/mL suspension by gavage, the Weiweishu decoction group was given 2.5 g/mL concentrated Chinese medicine decoction by gavage, and the normal group and the model group were given water by gavage, and the intervention lasted for 8 weeks.^[17–19]^

#### 2.3.4. Specimen collection

After the 8-week intervention period, the rats were fasted for 12 hours but were allowed to drink water. After anesthesia, a laparotomy was performed, blood was collected and centrifuged at 3000 r/min for 10 minutes, and the supernatant was collected for experiments. The entire stomach was excised, rinsed with normal saline, and cut into pieces. The antral portion of the stomach was individually fixed in a 4% paraformaldehyde solution (Biosharp, Beijing Lanjieke Technology Co., Ltd., Beijing, China, batch number 24029375). The remaining tissue was flash frozen with liquid nitrogen and stored in a −80°C freezer.

#### 2.3.5. Rat observation

The general condition of each group of rats, such as hair color, mental state, activity, stool characteristics, and weight, was observed.

#### 2.3.6. HE staining

The fixed gastric mucosal tissue was dehydrated, cleared, and embedded in paraffin. After embedding, the samples were continuously sectioned, stained with HE, and sealed. The morphology of the rat gastric mucosal tissue was observed under a microscope.

#### 2.3.7. ELISA kit

The levels of pepsinogen I/II (PG I/II) PGI, PGII, and TNF-α in the serum of rats in each group were measured according to the manufacturer’s instructions (batch numbers 202408 and M240523102a).

#### 2.3.8. *Fe^2+^, GSH, and MDA level detection*

To perform the experiment according to the corresponding kit instructions, the levels of iron ions, GSH, and SOD in the rats in each group were detected.

An iron assay kit was used to quantify the accumulation of Fe^2+^ in rat gastric tissue. Thirty milligrams of rat gastric tissue was collected, and 220 μL of iron assay buffer was added according to the kit instructions for lysis. The lysed tissues were homogenized via a tissue grinder (Tiss-24, Jingxin, Shanghai, China) at 4°C, incubated on ice for 25 minutes, and then centrifuged at 15,000×g for 16 minutes at 4°C. The supernatant was collected to determine the Fe^2+^ concentration.

A lipid oxidation malondialdehyde (MDA) kit (Beyotime, Shanghai Biyuntian Biotechnology Co., Ltd., Shanghai, China, #S0131S) was used to determine the total MDA activity in the gastric tissue. Gastric tissue (10 mg) was obtained and ground, and MDA sample preparation solution was added according to the kit instructions. The tissue was incubated on ice for 15 minutes. After incubation for 15 minutes, the tissue was centrifuged at 15,000×g for 10 minutes at 4°C. The supernatant was replenished and stored for MDA content determination.

A GSH/GSSG kit (Beyotime, Shanghai Biyuntian Biotechnology Co., Ltd. #S0053) was used to quantify the GSH levels in the rat gastric mucosal tissue. A total of 10 mg of rat stomach tissue was ground, 100 μL of M reagent was added, and the mixture was cooled on ice for 15 minutes. After centrifugation at 16000×g for 10 minutes at 4°C, the supernatant was collected and diluted to a certain extent according to the instructions, and the levels of GSH and GSSG were measured.

#### 2.3.9. Real time PCR

A total of 20 mg of rat gastric mucosal tissue was added to lysis buffer, and RNA was extracted from the gastric tissue lysate using an RNA kit (#R2072, Zimo RESEARCH). TRIzol (#15596026, Invitrogen) solution was added to the ground gastric tissue, which was subsequently lysed on ice for 30 minutes. The mixture was subsequently centrifuged at 12,000×g for 30 seconds, and the supernatant was discarded after centrifugation. The RNA was prewashed, and then, the RNA was washed with buffer sequentially. The above steps were repeated, the supernatant was discarded, and the sample was placed in the middle of the collection tube in the filter column. Nuclease-free water was added to elute the RNA. The concentration of RNA in rat gastric tissue was measured using a NanoDrop instrument, and then reverse transcription was performed to generate cDNA. SYBR Green Premix (Hunan Aikerui Biological Co., Ltd., Hunan, China, #AG11701) was used for qPCR preparation. Gene expression was detected via PCR (Bio-Rad, Hunan, China).

#### 2.3.10. Western blot analysis

A total of 20 mg of rat gastric mucosal tissue lysed on ice with lysis buffer (RIPA: protease inhibitor ratio of 150:1) for 30 minutes and centrifuged at 16,000×g for 10 minutes at 4°C. The supernatant was collected, and the protein concentration was measured using a BCA kit (#23235, Thermo Scientific). All the proteins were diluted to the same concentration after the protein concentration was calculated. After performing SDS–PAGE, the proteins were transferred to PVDF membranes via a semidry transfer method. The membranes were blocked with 5% skim milk at room temperature for 90 minutes, followed by 3 washes with TBST, each lasting 5 minutes. The samples were then incubated overnight at 4°C with antibodies against GPX4, MAPK, and HSP90AA1. Afterward, the membranes were washed 3 times with TBST for 5 minutes each, incubated with a secondary antibody at 37°C for 1 hour, and finally washed with TBST to develop the target protein bands in the dark.

#### 2.3.11. Statistical methods

All data are presented as mean ± standard deviation. Statistical analyses were performed using GraphPad Prism 9.5. Data were tested for normal distribution by Shapiro–Wilk test. Under the premise of a normal distribution, statistical significance was calculated by Student *t* test if only 2 groups were compared, and 1-way analysis of variance was employed if there were more than 2 groups. If they didn’t fit the normal distribution, the suitable nonparametric tests were used. Tukey’s test was employed for the post hoc test. A *P*-value <.05 was considered statistically significant.

## 3. Results

### 3.1. LC-MS/MS analysis of WWS

**Figure 1.**
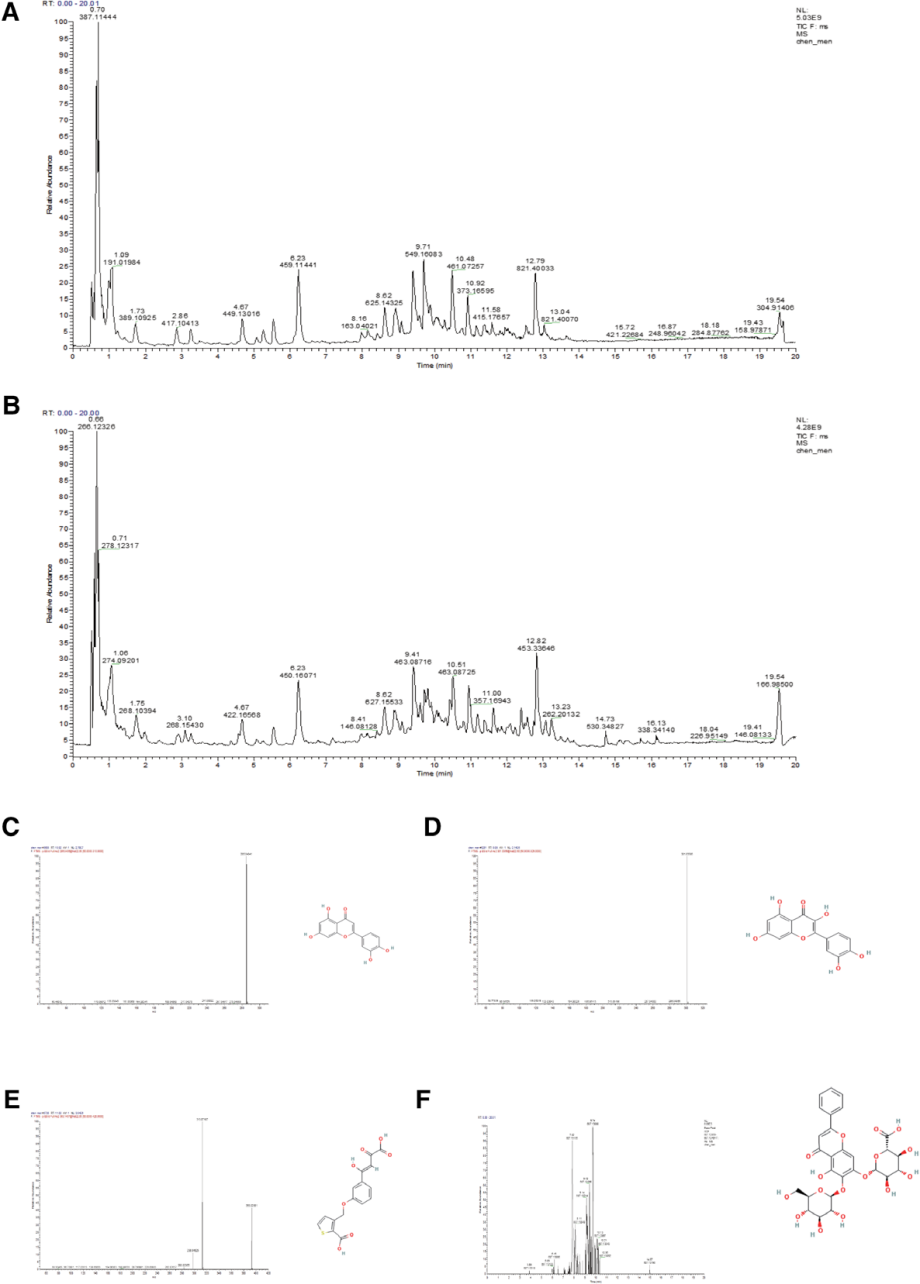
WWS total ion chromatogram (TIC) spectrum. (A) Positive ion mode; (B) negative ion mode; (C) secondary fragmentation and structural formula of luteolin; (D) secondary fragmentation and structural formula of quercetin; (E) secondary fragmentation and structural formula of formononetin; and (F) secondary fragmentation and structural formula of kaempferol. TIC = total ion chromatogram, WWS = Weiweisu.

The total ion chromatogram of WWS is presented in positive and negative ion modes (Fig. 1A, B). WWS resultantly 1017 absorbed compounds, and so on were identified based on characteristic fragmentation, neutral loss, and mass defect filter. The analysis found that its active ingredients such as luteolin, quercetin, kaempferol, formononetin, etc, and their secondary fragmentation was analyzed by liquid chromatography/mass spectrometry (LC/MS) and labeled with their structural formula, as shown in Figure 1C–F. Besides, we also built our own compounds-associated database concerning WWS based on TCMSP, HERB, and publicly available database.

### 3.2. Screening of Weiweisu decoction active ingredients and CAG-related targets

A total of 172 active ingredients were identified in WWS decoction through a screening process. Using the keyword “chronic atrophic gastritis,” thousands of targets were identified in the GeneCards database, the Online Mendelian Inheritance in Man database and the Therapeutic Target Database. Following a comprehensive analysis, 181 duplicate values were removed, resulting in the identification of a total of 2158 CAG-related targets.

### 3.3. Chinese herbal medicine-active ingredients-common targets network

**Figure 2.**
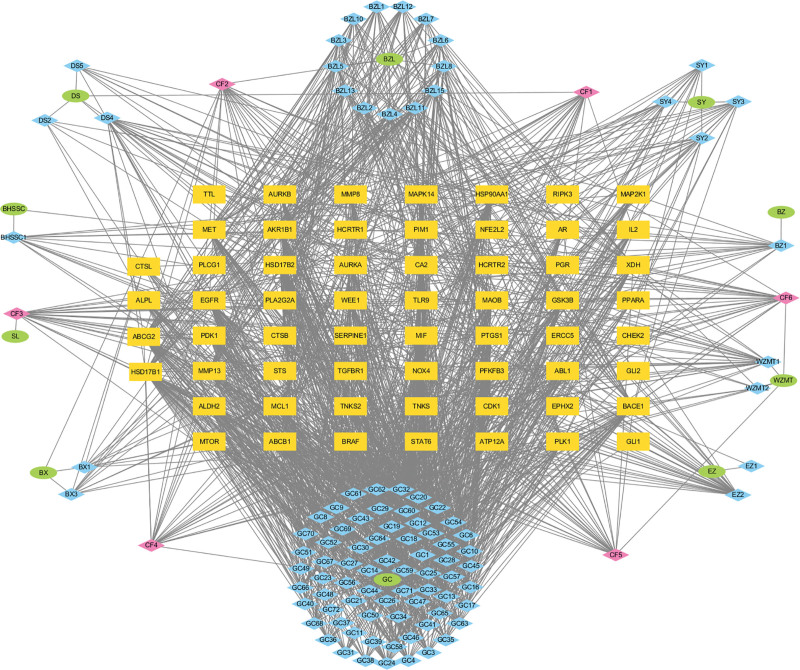
Network of herb-active ingredient-common targets. The ovals represent the herbs from the Weiweisu decoction; the rhombuses represent the active ingredients; the rectangles represent the common targets of the Weiweisu decoction and CAG. DS: *Codonopsis pilosula*; BZ: Atractylodes; BHSSC: *Oldenlandia diffusa*; SL: *Sparganii rhizoma*; EZ: Curcuma; BX: Pinellia; BZL: *Scutellaria barbata*; SY: *Chinese yam*; WZMT: *Ficus hirta* Vahl; GC: licorice. CF1: common components of Codonopsis pilosula and licorice; CF2: common components of Pinellia and *Scutellaria barbata*; CF3: common components of licorice and Sparganii Rhizoma; CF4: common components of Licorice and *Ficus hirta* Vahl; CF5: common components of *Ficus hirta* Vahl, *Scutellaria barbata* and Codonopsis pilosula; CF6: common components of *Ficus hirta* Vahl, *Scutellaria barbata*, licorice and Oldenlandia diffusa. CAG =chronic atrophic gastritis.

**Figure 3.**
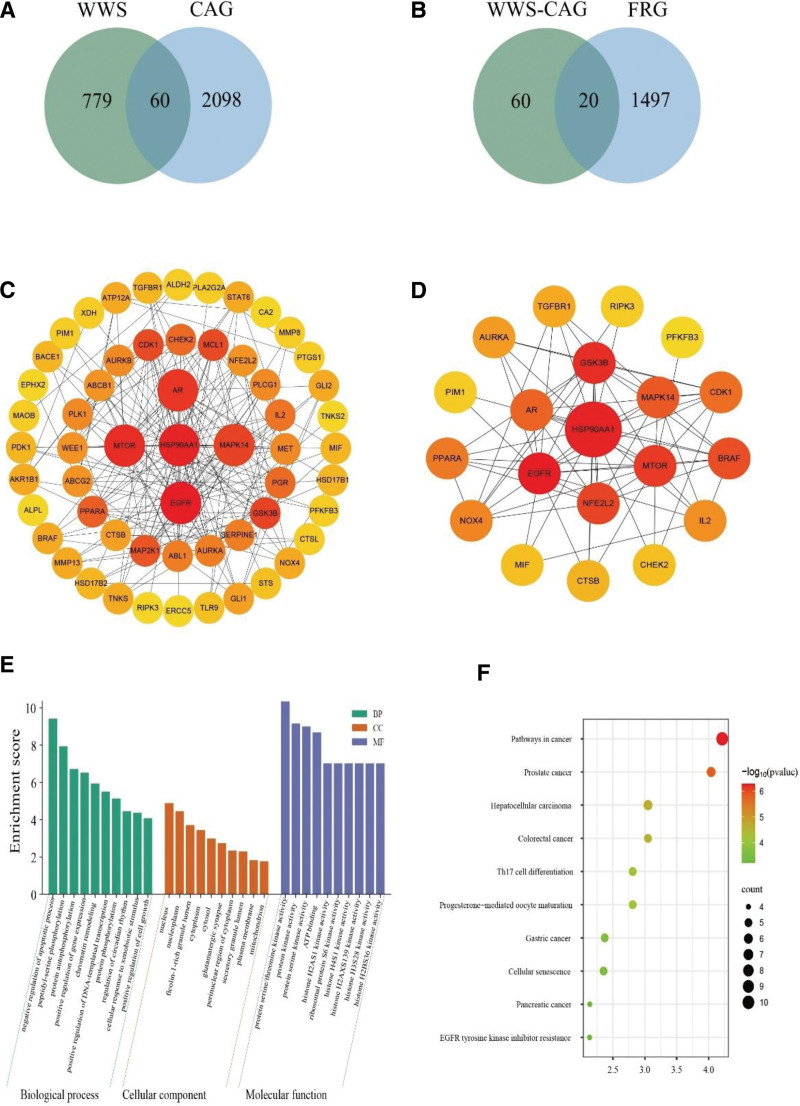
Network pharmacology results of Weiweisu decoction treatment for CAG. (A) Venn diagram of the identified target genes of Weiweisu decoction and CAG-related targets. (B) Venn diagram of the identified target genes of WWS-CAG and FRG targets. (C) PPI network of the common targets. (D) PPI network of the core targets. (E) GO enrichment analysis results. (F) Bubble chart illustrating the KEGG analysis results, showing the significance of different pathways. CAG = chronic atrophic gastritis, FRGs = erroptosis-related genes, GO = gene ontology, KEGG = Kyoto encyclopedia of genes and genomes, PPI = protein–protein interaction, WWS = Weiweisu.

Using Cytoscape 3.9.1 software, a visual network diagram of the common targets of WWS decoction and its active ingredients was constructed, as shown in Figure 2, namely the “traditional Chinese medicine-active ingredients-common targets” network diagram. The diagram contains 172 nodes and 1577 edges. CytoHubba plug-in analysis showed that the top 5 active ingredients were luteolin, breviscapine, baicalein, kaempferol and 7-methoxy-2-methyl isoflavone is shown in Table 1. Some information about these 5 active ingredients is shown in Table 3. These 5 compounds may represent the key active ingredients of gastroenterol decoction for the treatment of CAG. The 839 targets of WWS decoction were compared with 2158 targets related to CAG using the Venny 2.1.0 online website, and a Venn diagram was drawn. The results are shown in Figure 3A. Then, the WWS-CAG intersection targets were compared with ferroptosis targets and a Venn diagram was drawn. The results are shown in Figure 3B.

**Table 1:** Information on the 5 key active ingredients of the Weiweisu decoction granule.

MOL ID	Name	Degree	Relevant herbs
MOL000006	Luteolin	63	*Scutellaria barbata, Codonopsis pilosula, Ficus hirta* Vahl
MOL000392	Formononetin	42	*Sparganii Rhizoma,* Licorice
MOL002714	Baicalein	42	*Scutellaria barbata,* Pinellia
MOL000422	Kaempferol	40	*Ficus hirta* Vahl, Licorice
MOL003896	7-Methoxy-2-methyl isoflavone	40	*Codonopsis pilosula,* Licorice

**Table 2:** Docking results of core target proteins and key active ingredients.

Target name	PDB ID	Binding energy/(kcal/mol)				
		Luteolin	Formononetin	Baicalein	Kaempferol	7-Methoxy-2-methyl isoflavone
AR	2PIV	−8.8	−8.9	−9.1	−8.5	−9.0
EGFR	5UG9	−8.8	−8.5	−8.5	−8.5	−8.4
HSP90AA1	6OLX	−9.6	−9.8	−9.5	−10.2	−9.5
MAPK14	6SFO	−9.4	−8.7	−9.6	−9.4	−5.2
mTOR	3JBZ	−9.1	−8.6	−8.2	−8.8	−9.1

AR = androgen receptor, EGFR = epidermal growth factor receptor, MAPK = mitogen-activated protein kinase, mTOR = mammalian target of rapamycin.

**Table 3:** Primer sequences.

Species	Gene name	Direction	Primer sequences
Rat	mTOR	Forward	GGCAGGACGAGCGAGTGATG
		Reverse	CCGAGTTGGTGGACAGAGGAATG
	MAPK	Forward	CACGAGAATGTGATTGGTCTGTTGG
		Reverse	CACTTCACGATGTTGTTCAGGTCTG
	HSP90AA1	Forward	TGCGTATTTGGTTGCTGAGAAAGTG
		Reverse	ATTGGTTCACCTGTGTCTGTCCTC
	EGFR	Forward	ACTACGCCGCCTGCTTCAAG
		Reverse	CACTGTGCCAAATGCTCCTGAAC
	GAPDH	Forward	TGGAGAAACCTGCCAAGTATGATG
		Reverse	TATCCTTGCTGGGCTGGGTG

EGFR = epidermal growth factor receptor, MAPK = mitogen-activated protein kinase, mTOR = mammalian target of rapamycin.

### 3.4. Construction and analysis of the PPI network

The intersection targets of WWS and CAG and the intersection targets of WWS-CAG and ferroptosis-related genes were placed into the PPI network constructed by the STRING database. The TSV format file of the network was downloaded and imported into Cytoscape 3.9.0 for visualization. WWS and CAG are shown in Figure 3C. WWS-CAG and ferroptosis-related genes are shown in Figure 3D. In the figure, nodes represent targets and edges represent interactions between them. The size and color of nodes are related to their degree values: the larger the node and the darker the color, the higher the degree value. Targets with degree values, betweenness centrality and closeness centrality above the mean and median mean will be retained. The core targets finally determined include EGFR, heat shock protein HSP90AA1, mTOR, AR and MAPK 14, which may be the key targets of WWS decoction for the treatment of CAG involving anti-ferroptosis.

### 3.5. GO biological function and KEGG pathway enrichment analyses

After the DAVID website was used to perform GO analysis on the common targets, the 10 genes with the highest *P* values were selected and displayed in the form of a bubble chart, as shown in Figure 3E. The WWS decoction mainly regulates biological processes such as protein phosphorylation, gland development, positive regulation of transferase activity, and regulation of the stress response. The cellular components involved in the therapeutic effect include spindle microtubules, the cyst lumen, and the mitochondrial outer membrane. The treatment may involve or initiate molecular functions such as protein kinase and oxidoreductase activity, kinase binding, phosphatase binding, and transcription factor binding. The KEGG results were organized by *P* value, and the top 10 pathways were visualized as a bubble chart, as shown in Figure 3F. In this chart, the size of the bubbles represents the number of genes within each pathway, whereas the color indicates the *P* value: a deeper red signifies a smaller *P* value. These findings indicate that the Th17 cell differentiation process, and pathway in cancer may be critical pathways through which WWS decoction treats CAG and prevents its progression to GC.

### 3.6. Molecular docking and molecular dynamics simulation results

**Figure 4.**
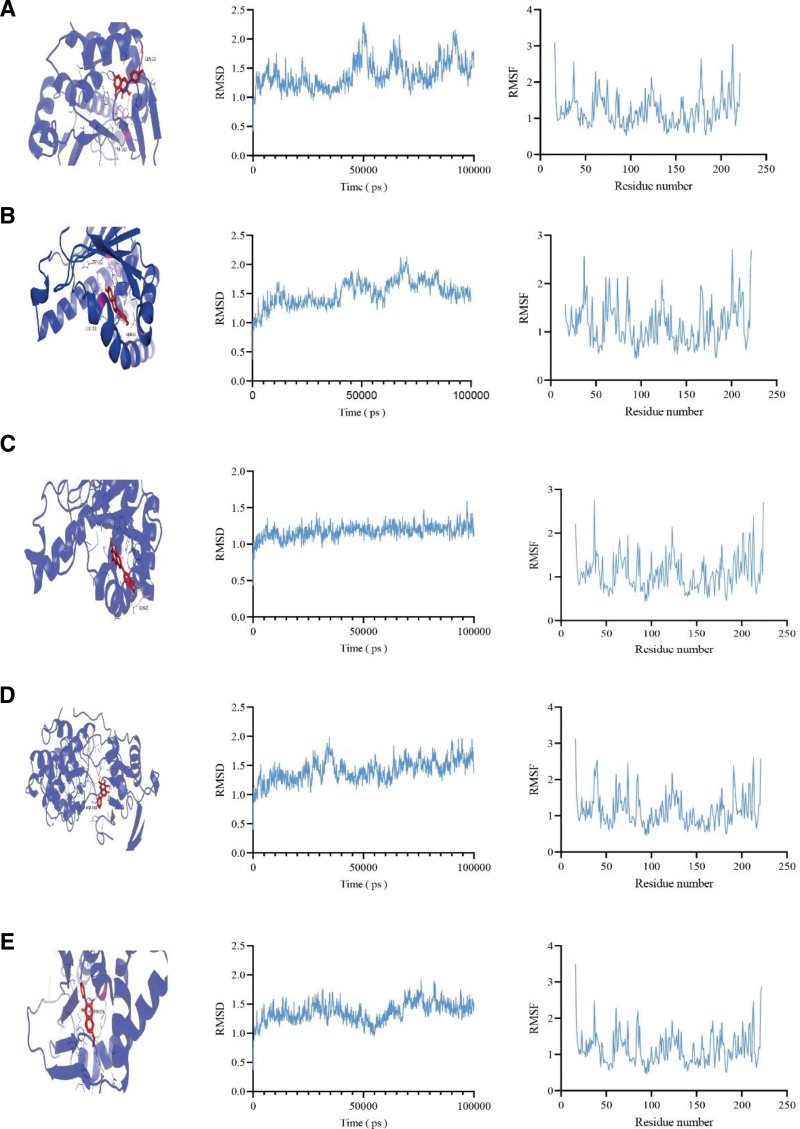
Molecular docking and molecular dynamics simulation of active ingredients and targets. (A) HSP90AA1 with kaempferol. (B) HSP90AA1 with formononetin. (C) HSP90AA1 with luteolin. (D) MAPK14 with baicalein. (E) HSP90AA1 with 7-methoxy-2-methyl isoflavone. MAPK = mitogen-activated protein kinase.

After screening the core targets on the basis of the PPI network and consulting the relevant literature, 5 components, namely, luteolin, formononetin, baicalein, kaempferol, and 7-methoxy-2-methyl isoflavone, were selected for molecular docking with EGFR, HSP90AA1, mTOR, AR, and MAPK. The results revealed that the binding affinities of luteolin, formononetin, baicalein, kaempferol, and 7-methoxy-2-methyl isoflavone to EGFR, HSP90AA1, mTOR, AR, and MAPK were all negative, and the average value was −8.868 kcal/mol, indicating that the active ingredients of the WWS decoction can strongly bind with targets related to CAG. The binding energy results are shown in Table 2, and a representative docking diagram with low binding energy is shown in Figure 4. To further verify the stability of the main target HSP90AA1 and the main active ingredient, we performed molecular dynamics simulations based on the high binding affinity observed in the molecular docking study. The simulation evaluated the equilibrium time and stability of the binding. The RMSD (root mean square deviation) value was used to evaluate the stability of the protein-ligand binding. The smaller the value, the less interference with the protein structure and the higher the stability. The root mean square fluctuation analysis evaluated the volatility of the protein-ligand complex. The smaller the value, the higher the stability. The energy curve visualizes the energy trend during the simulation and helps to further analyze the stability of the system.

### 3.7. General condition of the rats

**Figure 5.**
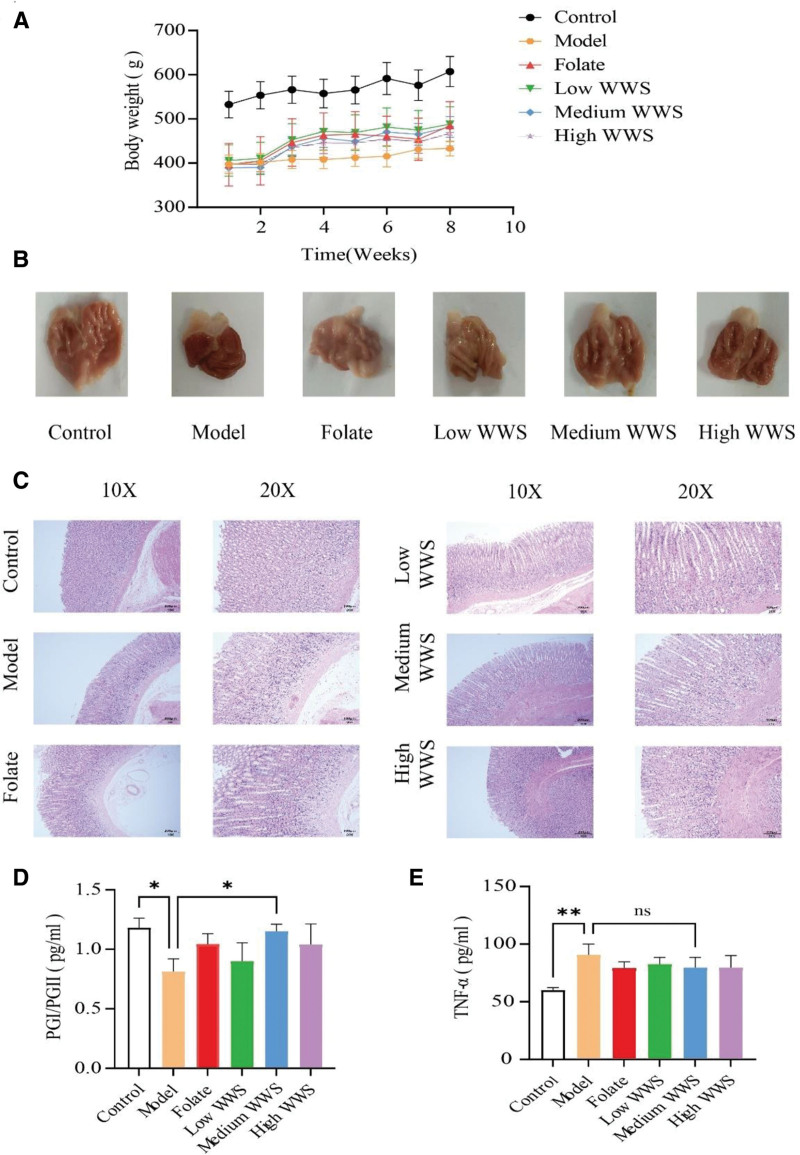
Pharmacological effects of Weiweisu decoction in CAG model rats. (A) Body weight gain curve during the treatment period. (B) Representative macroscopic images of gastric tissue morphology. (C) Hematoxylin and eosin (H&E) staining of gastric mucosa. (D) Serum PGI/PGII ratio and (E) serum TNF-a levels across treatment groups. Data are presented as mean ± standard deviation (SD), n = 3 per group. Statistical comparisons were performed using one-way ANOVA followed by Tukey’s post hoc test. *P < .05, **P < .01, ***P < .001. ANOVA = analysis of variance, CAG = chronic atrophic gastritis, H&E = hematoxylin and eosin, ns = no significant difference, PG I/II = pepsinogen I/II, SD = standard deviation.

After the model was established, the general condition of the rats was observed. The rats in the normal group were active, with clean and shiny hair, weight gain in line with their growth trends, normal stool characteristics and no special odor; the rats in the modeling group had stiff hair with a light yellow color, a small amount of soft stool, a with a smell. The model group rats exhibited reduced defecation compared with the normal group, and weighed less than the rats in the normal group did (Fig. 5A). Compared with those before treatment, the activity status, hair color, stool condition, weight, etc, of the rats in the folic acid group and WWS decoction group resembled those in the normal group.

### 3.8. Morphological analysis of the gastric tissue of the rats in each group

The gastric tissue of the rats in the normal group appeared light red and shiny, exhibiting normal morphology with well-defined mucosal folds. Microscopic examination revealed that the arrangement of the intrinsic glands in the gastric mucosa of the normal group of rats was not significantly abnormal. In contrast, the gastric tissue of the model group rats displayed signs of congestion, thinning, and fewer mucosal folds (Fig. 5B). Microscopic analysis of the model group revealed atrophy of the gastric mucosa, a sparse and disordered glandular arrangement, enlarged glandular cavities, inflammatory infiltration, and scattered hemorrhage. The findings from the gastric appearance, HE staining, and PGI/PGII analyses confirmed the successful construction of the CAG rat model, as shown in Figure 5C. After intervention with folic acid and WWS decoction, morphological abnormalities in the gastric mucosa were alleviated.

### 3.9. Serum PGI, PGII, and TNF-α levels in the rats in each group

The PGI/PGII content in the serum of the rats in the CAG model group was lower than that in the normal group, whereas the TNF-α content was greater (*P* < .05). Compared with that in the CAG model group, the PGI/PGII content in the WWS decoction dose groups and the folic acid group tended to increase, with the medium-dose WWS decoction group showing a more pronounced increase than the folic acid group. Moreover, the TNF-α content in the WWS decoction dose groups and the folic acid group was lower than that in the model group, with the medium-dose WWS decoction group demonstrating a greater decrease than the folic acid group did (*P* < .05). For further details, please refer to Figure 5D, E .

### 3.10. *Detection of Fe*^2+^*, GSH and MDA levels*

**Figure 6.**
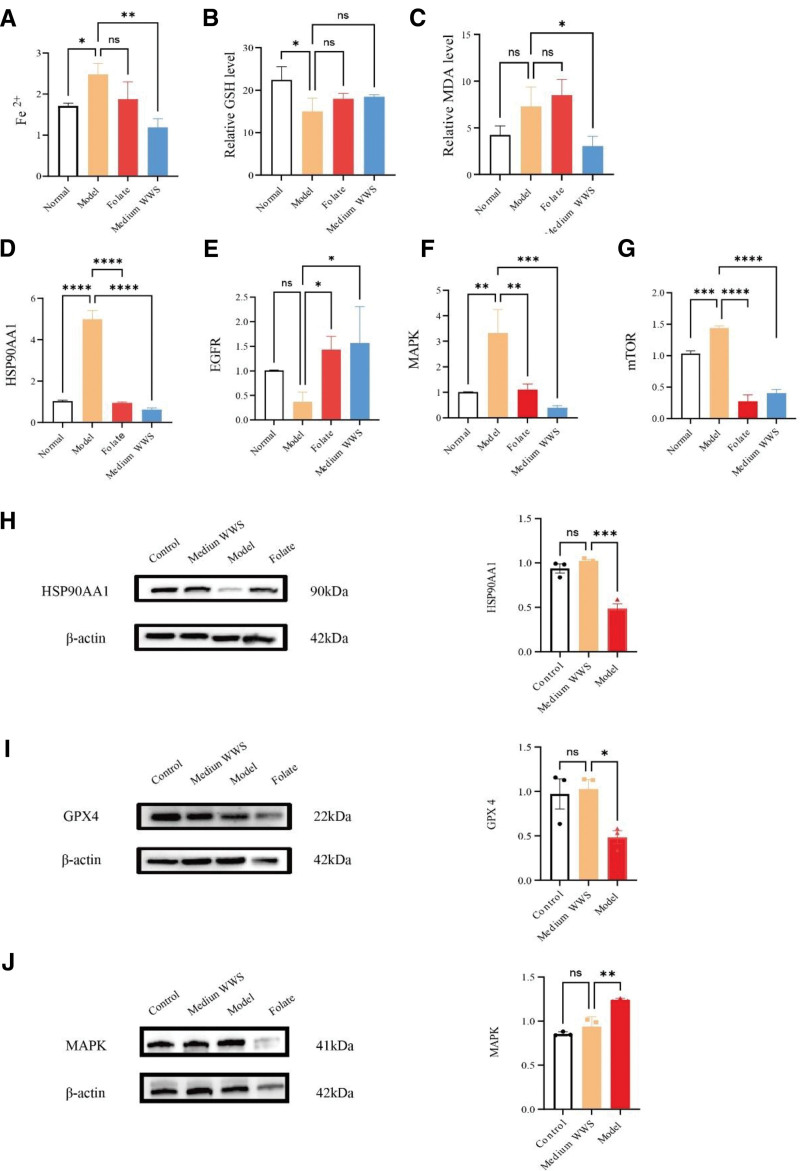
(A–D) mRNA expression levels of HSP90AA1, EGFR, MAPK, and mTOR measured by qRT-PCR. (E) Fe²⁺ concentration, (F) glutathione (GSH) level, and (G) malondialdehyde (MDA) level in gastric tissue. (H–J) Western blot analysis of HSP90AA1, GPX4, and MAPK protein levels in gastric mucosa. Quantification was performed using ImageJ. Data are expressed as mean ± SD (n = 3 per group). Statistical significance was determined using one-way ANOVA with Tukey’s multiple comparison test.*P < .05, **P < .01, ***P < .001, ****P < .0001; ns: not significant. ANOVA = analysis of variance, EGFR = epidermal growth factor receptor, GSH = glutathione, MAPK = mitogen-activated protein kinase, MDA = malondialdehyde, mTOR = mammalian target of rapamycin, qRT-PCR = quantitative real-time polymerase chain reaction, SD = standard deviation.

Compared with those in the normal group, the levels of Fe ions and MDA in the gastric mucosal tissues of the rats in the CAG model group were significantly greater. In contrast, the levels in the WWS decoction group tended to decrease relative to those in the model group. Additionally, the level of GSH in the gastric mucosal tissues of the rats in the CAG model group was lower than that in the normal group. Following treatment with folic acid and WWS decoction, the GSH levels tended to increase (Fig. 6A–C).

### 3.11. HSP90AA1, mTOR, MAPK, and EGFR mRNA expression

The mRNA levels of HSP90AA1, mTOR, and MAPK in the gastric mucosal tissues of the rats in the CAG model group were significantly greater than those in the normal group (*P* < .05), whereas the expression of EGFR was significantly lower (*P* < .05). Compared with those in the model group, the mRNA levels of HSP90AA1, mTOR, and MAPK in the folic acid group and the WWS decoction group were significantly lower (*P* < .05), whereas the expression of EGFR was significantly greater (*P* < .05). For further details, refer to Figure 6 D–G. The qPCR primer sequences can be found in Table 3.

### 3.12. HSP90AA1, GPX4, and MAPK protein expression

The protein expression levels of HSP90AA1 and GPX4 in the gastric mucosal tissues of the rats in the CAG model group were lower than those in the normal group (*P* < .05), whereas the protein expression of MAPK was greater (*P* < .05). After treatment with folic acid and WWS decoction, the protein expression levels of HSP90AA1 and GPX4 significantly increased (*P* < .05), and the protein expression level of MAPK significantly decreased, as shown in Figure 6H–J.

## 4. Discussion

GC remains one of the most prevalent malignancies worldwide, with a 5-year survival rate below 30% due to late-stage diagnosis. Although eradication of HP can reduce GC risk, current therapies often fall short in preventing mucosal atrophy and inflammation, underscoring the need for novel complementary approaches.^[20–22]^ From a TCM perspective, CAG corresponds to “Rupi syndrome,” characterized by spleen-qi deficiency, damp-heat, and blood stasis. WWS decoction, derived from Liu Junzi decoction and optimized for spleen-qi tonification and damp-heat resolution, demonstrated protective effects against CAG by targeting ferroptosis – a regulated form of cell death driven by iron accumulation and lipid peroxidation. Modern pharmacological studies have shown that Liujunzi decoction can regulate gastrointestinal peristalsis, eradicate HP infection, reduce mucosal inflammation caused by CAG, improve immune function and inhibit the growth of tumor cells, restore mitochondrial function and alleviate functional dyspepsia.^[23]^ Oldenlandia diffusa can inhibit inflammation and oxidative damage in gastric tissue^[24]^; the active components of Curcuma can inhibit the secretion of pro-inflammatory cytokines caused by HP^[25]^; the active components of Zhuru show potent and selective activity against the growth of GC cells^[26]^; and Chinese yam can protect against damage to the gastric mucosa,^[27]^ inhibit the growth of GC cells,^[28]^ and inhibit the inflammatory response associated with gastric ulcers.^[29]^

LC/MS analysis showed that the key active ingredients of the gastric decoction included quercetin, luteolin, formononetin, kaempferol, etc, which roughly overlapped with the main components of the network pharmacology analysis, luteolin, formononetin, baicalein, kaempferol, and 7-methoxy-2-methylisoflavone. Previous studies have shown that luteolin has bactericidal activity against HP antibiotic-resistant strains.^[30]^ Jafar M et al^[31]^ successfully developed gastric retention microsponges loaded with luteolin to prolong the antimicrobial activity of the drug in vivo, which showed the potential to eradicate HP infection, whereas Nam HH et al^[32]^ reported that the herb Huoxiang improved HCl/EtOH-induced gastritis by enhancing the effects of anti-inflammatory factors and inhibiting the infiltration of inflammatory cells and that this therapeutic activity was primarily mediated by its active ingredient luteolin. Mendonca MAA et al^[33]^ reported that formononetin improved inflammatory cell infiltration and edema and reduced gastric juice secretion. Additionally, formononetin inhibited the growth and migration of SGC7901 GC cells by targeting the Wnt/β-catenin and AKT/mTOR signaling pathways.^[34]^ Baicalein has anti-inflammatory and antioxidant functions and can effectively inhibit the effects of histamine on gastric secretion^[35]^; it can also inhibit the expression of HP infection-related genes and reduce the degree of gastric inflammation associated with HP infection.^[36]^ Kaempferol can reduce the levels of interleukin (IL)-6 and IL-1β by modulating the hedgehog signaling pathway, thereby aiding in the treatment of CAG^[37]^; moreover, Yeon MJ also demonstrated that kaempferol could inhibit the translocation of HP cytotoxin-associated gene A and vacuolating cytotoxin A to human GC epithelial cells, thereby reducing the expression of pro-inflammatory cytokines and exhibiting anti-inflammatory effects.^[38]^

Analysis of the nodes in the PPI network revealed 5 core targets, namely, EGFR, HSP90AA1, mTOR, AR, and MAPK14. Molecular docking analyses suggested that these targets could bind strongly with the key active ingredients, indicating that they were pivotal targets of WWS decoction granules in the treatment of CAG. EGFR, a member of the EGFR family, plays a crucial role in the physiological processes of cell growth, proliferation, and differentiation. Chaturvedi R et al^[39]^ reported that in patients with atrophic gastritis and HP infection, disease progression to IM or atypical hyperplasia was associated with increased levels of pEGFR and other genes in stomach samples. Chen X et al^[40]^ reported that palmatine, a quinoline alkaloid isolated from Coptis coptidis, could inhibit the MMP-10-dependent inflammatory response through the ADAM17/EGFR pathway, thereby alleviating HP-induced CAG. HSP90AA1 is a heat shock protein that is closely related to the occurrence and development of a variety of cancers. BaMC et al^[41]^ reported that ZNRD1-AS1 gene knockdown in GC tissues could inhibit the proliferation and metastasis of GC cells by targeting the miR-9-5p/HSP90AA1 axis. mTOR is a serine/threonine protein kinase that regulates cell growth, proliferation, and movement, thereby influencing protein synthesis and transcription. The mTOR inhibitor everolimus can reduce the oxidative stress caused by HP, significantly reduce the expression of pro-inflammatory cytokines, and thus protect gastric epithelial cells.^[42]^ The AR has recently been shown to play a role in the prevention of gastric metaplasia by inhibiting the activation of group 2 congenital lymphocytes.^[43]^ Moreover, MAPK14 is a member of the MAPK family that functions by regulating various physiological and pathological processes in cells, including cell growth and differentiation, adaptation to environmental stress, and the inflammatory response. Inhibition of the abnormal expression of MAPK signaling pathway genes can alleviate the inflammatory microenvironment in the stomach and protect the injured gastric mucosa, thereby improving gastric function, significantly reducing the number of regurgitations in rats with CAG, and preventing the development of HP-related gastritis.^[44–46]^

The results of the KEGG enrichment analysis indicated that the therapeutic effects of WWS decoction granules on CAG were primarily mediated through various cancer-related pathways, as well as pathways associated with Th17 cell differentiation and the HIF-1 signaling pathway. Th17 cells are a subset of helper T cells that primarily secrete IL-17, IL-22, and other pro-inflammatory factors and play a significant role in autoimmune diseases. These findings suggest that WWS decoction granules may exert an anti-inflammatory effect by inhibiting the differentiation of Th17 cells. Additionally, HIF-1 is known to contribute to cell death by promoting hypoxia and metabolic disorders. Notably, HIF-1α and HSP90 have been shown to interact in a pathway that mediates hypoxic signals. This interplay may further elucidate the mechanisms through which WWS decoction influences both inflammatory and cancer-related processes in CAG, potentially providing a multifaceted approach to treatment.^[47]^ Moreover, EGFR can activate the PI3K/Akt pathway and regulate glycolysis in the tumor microenvironment through HIF-1 under hypoxia^[48]^; it can also induce the phosphorylation of ERK, a member of the MAPK protein family, and then activate oncogenes to produce cancer cells.^[49]^ Inhibition of HIF-1 has a protective effect on gastritis caused by HP infection.^[50]^ Elhadidy MG et al^[51]^ reported that zeaxanthin inhibited the expression of HIF-1α through the PI3K/Akt/JNK signaling pathway and exerted gastroprotective effects against stress-induced gastritis. These findings suggest that the interactions between the pathway of cancer and the core targets identified in this study may be critical in the development of CAG and GC.

Both mRNA and Western blot analyses confirmed that the expression of MAPK in the model rat group was greater than that in the normal rat group. In contrast, compared with the model group, the WWS decoction group presented a downward trend in MAPK expression. These findings suggest that WWS decoction may treat CAG by modulating the MAPK signaling pathway. Additionally, GPX4 levels tended to decrease in the model group according to Western blot analysis, whereas those in the WWS decoction group tended to increase. The analysis of iron ions, GSH, and MDA revealed that iron and GSH levels were increased in the model group but decreased in the treatment group. These findings suggest that the therapeutic effects of WWS decoction on CAG may be related to the process of ferroptosis. Further molecular docking identified the core target heat shock protein HSP90AA1 with the highest binding affinity. Molecular dynamics simulation revealed that the binding affinity and stability of HSPAA1 with the main active ingredients of WWS did not change over time and fluctuated greatly. Experimental verification showed that the expression of HSP90AA1 showed a downward trend in Western blot analysis. Therefore, we speculate that WWS decoction may exert its therapeutic effects on CAG by downregulating members of the HSP90AA1, MAPK targets.

## 5. Conclusion

This study systematically elucidated the pharmacological basis of WWS decoction in treating CAG by integrating LC-MS/MS-based compound identification, network pharmacology, molecular docking, and in vivo validation. The results suggest that WWS exerts protective effects on gastric mucosa by modulating ferroptosis-related targets such as HSP90AA1 and MAPK, potentially through cancer-related signaling pathways. These findings offer new mechanistic insights into the multicomponent, multi-target therapeutic potential of WWS against CAG and its precancerous progression. Nevertheless, this study has several limitations. First, the relatively small sample size and lack of formal power calculation may limit the statistical strength of the findings. Second, although our results suggest potential anti-ferroptotic effects of WWS decoction in CAG, further studies with longer observation periods, cytotoxicity evaluations, safety evaluations, dose–response assessments, and the inclusion of ferroptosis-specific inhibitors are needed to confirm the precise mechanisms and clinical relevance.

## Author contributions

**Conceptualization:** Shenglan Wu, Yubo Jin.

**Data curation:** Meng Chen, Yan Dang.

**Formal analysis:** Meng Chen, Jialin Shen.

**Funding acquisition:** Meng Chen, Jie Liu, Caiqun Bie.

**Investigation:** Meng Chen.

**Methodology:** Xiaoying Zhu.

**Software:** Hua Chen, Jialin Xu.

**Writing – original draft:** Meng Chen.

## Supplementary Material


