# Supplementary material for: Deciphering the pharmacological mechanisms of Weiweisu decoction in chronic atrophic gastritis: Insights from network pharmacology, molecular dynamics, and in vivo validation
Source: Medicine (Baltimore). 2025 Nov 7;104(45):e45348. doi: 10.1097/MD.0000000000045348 (PMC12599655; doi:10.1097/MD.0000000000045348)
Supplement: Supplementary file 1 [file medi-104-e45348-s001.pdf]

Table S1：Relevant database and software in this study.

Number	Name	Vers-ion	Website
1	TCMSP	2.3	https://old.tcmsp-e.com/tcmsp.php
2	TCMID	1.0	http://www.megabionet.org/tcmid/
3	HERB	-	http://herb.ac.cn/
4	CNKI	-	https://www.cnki.net/
5	Pubmed	-	https://pubmed.ncbi.nlm.nih.gov/
6	Pubchem	-	https://pubchem.ncbi.nlm.nih.gov/
7	SwissTargetADME	-	http://www.swissadme.ch/
8	SwissTargetPrediction	-	http://www.swisstargetprediction.ch/
9	Uniprot	-	https://www.uniprot.org/
10	OMIM	-	https://omim.org/
11	GeneCards	5.12	https://www.genecards.org/
12	TTD	-	http://db.idrblab.net/ttd/
13	Venny	2.1.0	https://bioinfogp.cnb.csic.es/tools/venny/
14	Cytoscape	3.9.1	https://cytoscape.org/
15	STRING	11.5	https://cn.string-db.org/
16	DAVID	104.0	https://david.ncifcrf.gov/
17	imageGP	-	http://www.ehbio.com/ImageGP/index.php/
18	RCSB	-	https://www.pdbus.org/
19	AutoDock	4.0	https://autodock.scripps.edu/
20	AutoVina	4.0	https://vina.scripps.edu/
21	PyMOL	2.4	https://pymol.org/2/

Table S2: Partial information of active ingredients.

MOL ID	Compound	OB (%)	DL	Relevant herbs	Corresp-onding number in network
MOL003896	7-Methoxy-2-methyl isoflavone	42.56	0.2	Codonopsis pilosula, Licorice	CF1
MOL002714	baicalein	33.52	0.21	Banxia, Scutellaria barbata	CF2
MOL000392	formononetin	69.67	0.21	Licorice, Sparganii Rhizoma	CF3
MOL000422	kaempferol	41.88	0.24	Licorice, Ficus hirta Vahl	CF4
MOL000006	luteolin	36.16	0.25	Scutellaria barbata, Codonopsis	CF5

MOL000098	quercetin	46.43	0.28	pilosula, Ficus hirta Vahl Oldenlandia diffusa, Scutellaria barbata, Licorice, Ficus hirta Vahl	CF6
MOL001670	2-methoxy-3-methyl-9,10-anthraquinone	37.83	0.21	Oldenlandia diffusa	BHSSC1
MOL002670	Cavidine	35.64	0.81	Pinellia	BX1
MOL006957	(3S,6S)-3-(benzyl)-6-(4-hydroxybenzyl)piperazine-2,5-quinone	46.89	0.27	Pinellia	BX3
MOL000072	8β-ethoxy atractylenolide Ⅲ	35.95	0.21	Atractylodes	BZ1
MOL001040	(2R)-5,7-dihydroxy-2-(4-hydroxyphenyl)chroman-4-one	42.36	0.21	Scutellaria barbata	BZL1
MOL012245	5,7,4'-trihydroxy-6-methox-yflavanone	36.63	0.27	Scutellaria barbata	BZL2

MOL012246	5,7,4'-trihydroxy-8-methox-yflavanone	74.24	0.26	Scutellaria barbata	BZL3
MOL012248	5-hydroxy-7,8-dimethoxy-2-(4-methoxyphenyl)chromone	65.82	0.33	Scutellaria barbata	BZL4
MOL012250	7-hydroxy-5,8-dimethoxy-2-phenyl-chromone	43.72	0.25	Scutellaria barbata	BZL5
MOL012251	Chrysin-5-methylether	37.27	0.2	Scutellaria barbata	BZL6
MOL012266	rivularin	37.94	0.37	Scutellaria barbata	BZL7
MOL000173	wogonin	30.68	0.23	Scutellaria barbata	BZL8
MOL002719	6-Hydroxynaringenin	33.23	0.24	Scutellaria barbata	BZL10
MOL002915	Salvigenin	49.07	0.33	Scutellaria barbata	BZL11
MOL000351	Rhamnazin	47.14	0.34	Scutellaria barbata	BZL12
MOL005190	eriodictyol	71.79	0.24	Scutellaria barbata	BZL13

MOL008206	Moslosooflavone	44.09	0.25	Scutellaria barbata	BZL15
MOL005321	Frutinone A	65.9	0.34	Codonopsis pilosula	DS2
MOL008400	glycitein	50.48	0.24	Codonopsis pilosula	DS4
MOL008411	11-Hydroxyrankinidine	40	0.66	Codonopsis pilosula	DS5
MOL000906	wenjine	47.93	0.27	Curcuma	EZ1
MOL000940	bisdemethoxycurcumin	77.38	0.26	Curcuma	EZ2
MOL001484	Inermine	75.18	0.54	Licorice	GC1
MOL000239	Jaranol	50.83	0.29	Licorice	GC3
MOL000354	isorhamnetin	49.6	0.31	Licorice	GC4
MOL000417	Calycosin	47.75	0.24	Licorice	GC6
MOL000497	licochalcone a	40.79	0.29	Licorice	GC8
MOL000500	Vestitol	74.66	0.21	Licorice	GC9
MOL001792	DFV	32.76	0.18	Licorice	GC10
MOL002311	Glycyrol	90.78	0.67	Licorice	GC11
MOL002565	Medicarpin	49.22	0.34	Licorice	GC12
MOL002844	Pinocembrin	64.72	0.18	Licorice	GC13
MOL003656	Lupiwighteone	51.64	0.37	Licorice	GC14
MOL004328	naringenin	59.29	0.21	Licorice	GC16

MOL004808	glyasperin B	65.22	0.44	Licorice	GC17
MOL004810	glyasperin F	75.84	0.54	Licorice	GC18
MOL004811	Glyasperin C	45.56	0.4	Licorice	GC19
MOL004814	Isotrifoliol	31.94	0.42	Licorice	GC20
MOL004815	(E)-1-(2,4-dihydroxyphenyl)-3-(2,2-dimethylchromen-6-yl)prop-2-en-1-one	39.62	0.35	Licorice	GC21
MOL004820	kanzonols W	50.48	0.52	Licorice	GC22
MOL004824	(2S)-6-(2,4-dihydroxyphenyl)-2-(2-hydroxypropan-2-yl)-4-methoxy-2,3-dihydrofuro[3,2-g]chromen-7-one	60.25	0.63	Licorice	GC23
MOL004828	Glepidotin A	44.72	0.35	Licorice	GC24
MOL004833	Phaseolinisoflavan	32.01	0.45	Licorice	GC25
MOL004835	Glypallichalcone	61.6	0.19	Licorice	GC26
MOL004838	8-(6-hydroxy-2-benzofuran-yl)-2,2-dimethyl-5-9-chromenol	58.44	0.38	Licorice	GC27
MOL004841	Licochalcone B	76.76	0.19	Licorice	GC28

MOL004849	3-(2,4-dihydroxyphenyl)-8-(1,1-dimethylprop-2-enyl)-7-hydroxy-5-methoxy-coumarin	59.62	0.43	Licorice	GC29
MOL004855	Licoricone	63.58	0.47	Licorice	GC30
MOL004856	Licoricenin A	51.08	0.4	Licorice	GC31
MOL004857	Licoricenin B	48.79	0.45	Licorice	GC32
MOL004863	3-(3,4-dihydroxyphenyl)-5,7-dihydroxy-8-(3-methylbut-2-enyl)chromone	66.37	0.41	Licorice	GC33
MOL004864	5,7-dihydroxy-3-(4-methoxyphenyl)-8-(3-methylbut-2-enyl)chromone	30.49	0.41	Licorice	GC34
MOL004866	2-(3,4-dihydroxyphenyl)-5,7-dihydroxy-6-(3-methylbut-2-enyl)chromone	44.15	0.41	Licorice	GC35
MOL004879	Glycyrin	52.61	0.47	Licorice	GC36
MOL004882	Licocoumarone	33.21	0.36	Licorice	GC37
MOL004883	Licoisoflavone	41.61	0.42	Licorice	GC38
MOL004884	Licoisoflavone B	38.93	0.55	Licorice	GC39
MOL004885	licoisoflavanone	52.47	0.54	Licorice	GC40
MOL004891	shinpterocarpin	80.3	0.73	Licorice	GC41

MOL004898	(E)-3-[3,4-dihydroxy-5-(3-methylbut-2-enyl)phenyl]-1-(2,4-dihydroxyphenyl)prop-2-en-1-one	46.27	0.31	Licorice	GC42
MOL004904	licopyranocoumarin	80.36	0.65	Licorice	GC43
MOL004907	Glyzaglabrin	61.07	0.35	Licorice	GC44
MOL004908	Glabridin	53.25	0.47	Licorice	GC45
MOL004910	Glabranin	52.9	0.31	Licorice	GC46
MOL004911	Glabrene	46.27	0.44	Licorice	GC47
MOL004912	Glabrone	52.51	0.5	Licorice	GC48
MOL004913	1,3-dihydroxy-9-methoxy-6-benzofurano[3,2-c] chromenone	48.14	0.43	Licorice	GC49
MOL004914	1,3-dihydroxy-8,9-dimethoxy-6-benzofurano[3,2-c] chromenone	62.9	0.53	Licorice	GC50
MOL004915	Eurycarpin A	43.28	0.37	Licorice	GC51
MOL004924	(-)-Medicocarpin	40.99	0.95	Licorice	GC52
MOL004935	Sigmoidin-B	34.88	0.41	Licorice	GC53

MOL004941	(2R)-7-hydroxy-2-(4-hydroxyphenyl)chroman-4-one	71.12	0.18	Licorice	GC54
MOL004945	(2S)-7-hydroxy-2-(4-hydroxyphenyl)-8-(3-methylbut-2-enyl)chroman-4-one	36.57	0.32	Licorice	GC55
MOL004948	Isoglycyrol	44.7	0.84	Licorice	GC56
MOL004949	Isolicoflavonol	45.17	0.42	Licorice	GC57
MOL004957	HMO	38.37	0.21	Licorice	GC58
MOL004959	1-Methoxyphaseollidin	69.98	0.64	Licorice	GC59
MOL004961	Quercetin der.	46.45	0.33	Licorice	GC60
MOL004966	3'-Hydroxy-4'-O-Methylglabridin	43.71	0.57	Licorice	GC61
MOL004974	3'-Methoxyglabridin	46.16	0.57	Licorice	GC62
MOL004978	2-[(3R)-8,8-dimethyl-3,4-dihydro-2H-pyrano[6,5-f]3-chromen-3-yl]-5-methoxy-phenol	36.21	0.52	Licorice	GC63
MOL004980	Inflacoumarin A	39.71	0.33	Licorice	GC64
MOL004990	7,2',4'-trihydroxy－5-methoxy-3－arylcoumarin	83.71	0.27	Licorice	GC65
MOL004991	7-Acetoxy-2-methylisoflavone	38.92	0.26	Licorice	GC66

MOL005000	Licoricenin G	60.44	0.39	Licorice	GC67
MOL005003	Licoagrocarpin	58.81	0.58	Licorice	GC68
MOL005008	Glycyrrhiza flavonol A	41.28	0.6	Licorice	GC69
MOL005012	Licoagroisoflavone	57.28	0.49	Licorice	GC70
MOL005016	Odoratin	49.95	0.3	Licorice	GC71
MOL005017	Phaseol	78.77	0.58	Licorice	GC72
MOL001559	piperlonguminine	30.71	0.18	Chinese yam	SY1
MOL000310	Denudatin B	61.47	0.38	Chinese yam	SY2
MOL000322	Kadsurenone	54.72	0.38	Chinese yam	SY3
MOL005429	hancinol	64.01	0.37	Chinese yam	SY4
MOL001689	acacetin	34.97	0.24	Ficus hirta Vahl	WZMT1
MOL001945	bergapten	42.21	0.13	Ficus hirta Vahl	WZMT2
